# Should I Stay or Should I Go – Cognitive Conflict in Multi-Attribute Signals Probed with East and West German ‘Ampelmännchen’ Traffic Signs

**DOI:** 10.1371/journal.pone.0064712

**Published:** 2013-05-24

**Authors:** Claudia Peschke, Bettina Olk, Claus C. Hilgetag

**Affiliations:** 1 School of Humanities and Social Sciences, Jacobs University Bremen, Bremen, Germany; 2 Department of Computational Neuroscience, University Medical Center Hamburg-Eppendorf, Hamburg, Germany; 3 Department of Health Sciences, Boston University, Boston, Massachusetts, United States of America; Cardiff University, United Kingdom

## Abstract

In post-unification Germany, lingering conflicts between East and West Germans have found some unusual outlets, including a debate of the relative superiority of East and West German ‘Ampelmännchen’ pedestrian traffic signs. In our study, we probed the visual efficacy of East and West German Ampelmännchen signs with a Stroop-like conflict task. We found that the distinctive East German man-with-hat figures were more resistant to conflicting information, and in turn produced greater interference when used as distractors. These findings demonstrate Stroop-like effects for real-life objects, such as traffic signs, and underline the practical utility of an East German icon.

## Introduction

Efficient selection of attentional focus and appropriate behavioral responses are crucial when relevant and irrelevant pieces of information compete for mental representation [1]. Conversely, distraction or improper selection of responses can have highly detrimental consequences, for instance when navigating through dense and fast city traffic. Our attention and behavior in such complex situations is guided by signals with multiple attributes. For instance, pedestrian traffic lights in many countries communicate stop/go signals by color (red/green) as well as shape (standing/walking figure), but also by words (Walk/Don’t Walk) or sounds (saying “Wait” or a bleep). Frequently, the particular form of these signals is not only motivated by functional requirements, but also reflects regional aesthetic preferences and traditions.

**Figure 1:**
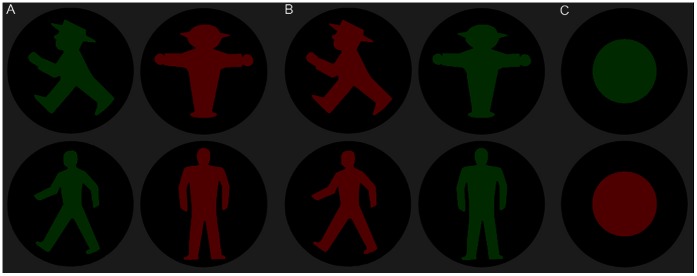
Ampelmännchen stimuli. A) Congruent condition: go/stop signs from East (upper panel) and West Germany (lower panel); left figures signaling “go”; right figures “stop”; B) Incongruent condition: go/stop signs with altered colors; C) Examples for control condition: color circles with same area size (number of pixels) of color as corresponding signs.

Interestingly, in Germany, two different versions of ‘Ampelmännchen’ pedestrian traffic signs exist that originated from two different political systems, the former German Democratic Republic and the Federal Republic of Germany. In 1961, the traffic psychologist Karl Peglau invented the East German Ampelmännchen in response to increased numbers of traffic accidents. In order to reduce confusion by shared signs, he suggested installing specific traffic lights for each kind of road user [2], [3]. For pedestrian signs, he created red and green standing and walking figures, combining color and shape information. The combination of both features was intended to facilitate and improve the visual and cognitive perception of the signals, but also to increase the acceptance of the signals [2], [3]. Thus, the East Ampelmännchen was designed to be emotionally appealing, especially to pedestrians at risk, such as children and the elderly. By contrast, a more abstract and neutral Ampelmännchen design was introduced in West Germany (see Figure 1A). After German unification in 1990, the two Ampelmännchen versions triggered a debate of the relative superiority of the East or West German Ampelmännchen. Specifically, the old-fashioned East German man-with-hat figure was claimed to have high visual efficacy [4], and it has become an object of affection and ‘ostalgia’ 20 years after the country’s unification: “East German opposition to the relentless Westernization […] was articulated in a well-publicized campaign to save the cute, jauntily-hatted ‘little lamp man’ on GDR traffic lights from being replaced by his characterless West German counterpart” (cf. [5], p. 222; see also [6]). Eventually, the general replacement of East by West German Ampelmännchen signs was stopped, and in more recent years the East version has also been introduced in some West German cities [7].

Against the background of this political and cultural debate, it is intriguing to assess the relative efficacy of different attributes of multi-feature traffic signals, particularly their characteristic shape. Specifically, to examine the visual efficacy of East and West Ampelmännchen signs, we probed them with a Stroop-like conflict task. In the classic Stroop task [8], [9], color words are presented in ink colors that are congruent or incongruent with the words’ semantic content. Characteristically, subjects respond less efficiently in the incongruent condition when naming the ink color, due to the highly automated skill of word reading that interferes with color naming [10].

Here we presented East and West Ampelmännchen signs either in their original (congruent) or alternative (incongruent) color and asked participants to make stop/go responses based on sign color or shape. One aim of the study was to establish whether the real-life objects could induce interference effects similar to color and color words in the original Stroop task. It is reasonable to assume that incongruent color and shape combinations of traffic signs lead to a poorer performance than congruent combinations, since the typical meaning of the figures’ colors and shapes is well established and learned and thus strongly linked to behavioral responses. Therefore, an incongruent feature combination should interfere with the processing of the relevant feature. However, it is not clear whether color or shape has a stronger impact on performance. The traditional Stroop task only reports a conflict in the incongruent condition when naming the ink color but not reading the word, due to the automated processing of words [10]. In the present study, on the one hand, the meaning of the traffic light colors might be more overlearned than the meaning of the shape. On the other hand, the shape of the figures is more self-explanatory. Thus, it is not clear which of the stimulus features may lead to a stronger interference effect or whether there is a difference between them at all. Moreover, the study aimed at examining differences in the efficacy of the different Ampelmännchen variants. Simply speaking, is the East Ampelmännchen only cute or also more visually effective? We hypothesized a better performance for East than West signs when participants had to respond to the shape of the figures, and a stronger interference of incongruent East compared to West signs when color responses were required. The reason for that assumption was the potentially stronger expressiveness of the East Ampelmännchen due to the characteristic design of the figures.

In summary, we investigated (i) if real-life pedestrian traffic signs induced Stroop-like interference when shape and color were incongruent, (ii) which attribute, color or shape, produced more interference, and (iii) whether there were differences in interference produced by the West and East variants of the Ampelmännchen signs.

## Methods

### Participants

The study was performed in accordance with the guidelines of the Declaration of Helsinki and of the German Psychological Society. An ethical approval was not required since the study did not involve any risk or discomfort for the participants. All participants were informed about the purpose and the procedure of the study and gave written informed consent prior to the experiment. All data were analyzed anonymously.

Twenty right-handed participants (mean age 20.7 years, 8 females) from different countries participated. Ten participants reported familiarity with the West signs only, one with the East signs only, eight with both signs and one with none of the figures. None of the participants had an impairment of color vision. Participants were compensated for taking part in the study.

### Stimuli and Procedure

As stimuli we used stop/go signs adapted from the original East and West German traffic-light displays for pedestrians. To control for contrast and color saliency, colors were equally darkened and made isoluminant, using the Gimp software (www.gimp.org). Height/width of the stimuli was 15.1°. The signs were either presented in the original, congruent color (i.e., go - green; stop - red) or in the incongruent color (go - red; stop – green; see Figure 1). The combination of color (red/green), shape (stop/go) and variant (East/West) resulted in eight different stimuli. Differing sizes of colored spaces in the East/West signs were accounted for by eight circular control stimuli containing the same number of color pixels as the respective signs. Stimuli were presented centrally on a TFT monitor (42.5 cm viewing distance) using Presentation software (www.neurobs.com/presentation). Head position was supported with a chin and forehead rest. Room lighting was kept constant (265 lx).

The experiment consisted of three different tasks. (i) In the **color task**, participants were instructed to respond to the sign color (green or red). (ii) In the **shape task**, participants were asked to respond to the shape of the figure (go or stop). (iii) In the **control task**, participants had to respond to the color of the circular control stimuli (green or red). Participants were instructed to respond as fast as possible, pressing the left or right button of a keyboard with their right-hand index or middle finger (e.g. left button for green or walking figures). Assignments of buttons to response were counterbalanced across participants.

For the color and shape tasks, respectively, each of the eight traffic sign stimuli was presented 30 times, resulting in 240 trials/task. For the control task, the respective circular control stimuli were also presented 30 times each. To avoid a decrease of performance due to fatigue, each of the three tasks was divided into three blocks (of 80 trials) that were performed in a row with short breaks inbetween. The trial order within a block was randomized, with the restriction that there was no iteration of the same stimulus in consecutive trials. Task order was counterbalanced across participants, with the control task always in the middle. In each trial, a visual stimulus was presented until response or for a maximum of 2700 ms, whichever came first. Response button and response latency were recorded by the presentation software.

### Data Analysis

We calculated the percentage of errors per participant and type of trial. Median reaction time of all correct trials was computed for each participant and type of trial separately. Since accuracy and latency often interrelate, they were combined into a single variable, effective reaction time: RT* = latency/accuracy [11], thus, avoiding a potential speed-accuracy trade-off and creating a unified measure for the performance of the participants. Performance was analyzed by repeated measures analyses of variance (ANOVA) and post-hoc paired-samples t-tests.

## Results

Across conditions, participants reached a high level of accuracy with a group mean of 97.1% correct responses (range: 92.2–99.7%; *SD*: 2.1). One-way ANOVAs did not show a significant difference in accuracy neither between tasks (*F*(2,18) = 0.4, *p*>.05, η*_p_^2^* = .04; color task: 97.0%; shape task: 97.0%; control task: 97.4%) nor between variants (*F*(1,19) = 1.8, *p*>.05, η*_p_^2^* = .09; East sign: 97.1%; West sign: 96.7%). Given the consistently high accuracy of responses, results for *RT* and RT* were largely identical, except that interactions for simple *RT* in some cases were only marginally significant, see below. For further analyses we focused on the effective reaction time.

In order to exclude potential effects resulting from the differing sizes of colored spaces in the East and West signs, we first analyzed performance in the control condition. A paired-samples t-test between the control stimuli with same color area sizes as the East (517 ms, *SD* = 62) and West sign variants (519 ms, *SD* = 67) did not show a significant difference in RT*, *t*(19) = 0.5, *p*>.05, *d* = 0.12, indicating that observed differences in the color and shape task were not a result of different image sizes between the variants. Thus, control stimuli were not analyzed further.

**Figure 2:**
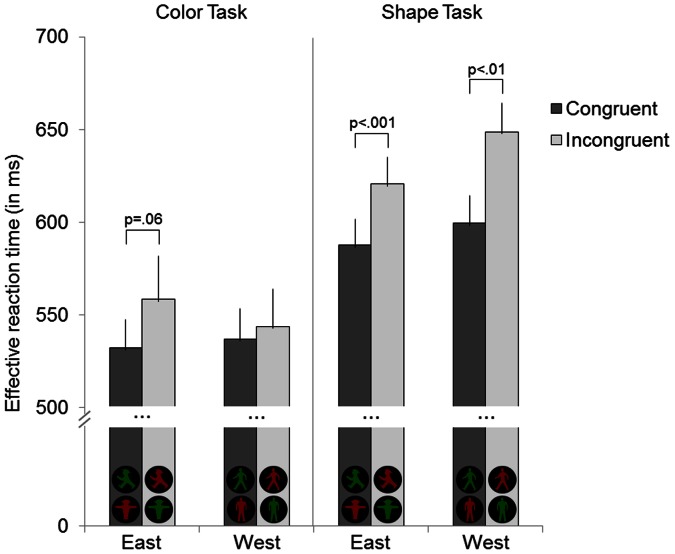
Effective reaction time (RT*) depending on task and stimulus conditions. RT* was longer for the shape than color task, incongruent than congruent stimuli, and West compared to East signs. Error bars represent standard errors.

To assess whether Stroop-like interference occurred and was modulated by task and variant of the Ampelmännchen signs, a repeated-measures ANOVA with the factors **congruency** (congruent/incongruent), **task** (color/shape) and **variant** (East/West) was computed and supplemented by paired-samples t-tests. For a graphic illustration of the results see Figure 2 (for mean *RT*s* and *SD*s see Table 1).

**Table 1:** Mean effective reaction time and *SD* (in ms) for different conditions.

	Color task					Shape task				
	East sign		West sign		East sign			West sign		
	Mean	*SD*	Mean	*SD*	Mean		*SD*	Mean	*SD*	
**Congruent**	532	69	537	74	588		64	599	67	
**Incongruent**	558	105	544	91	621		65	649	71	

Significant main effects of congruency, *F*(1,19) = 33.7, *p*<.001, η*_p_^2^* = .64; task, *F*(1,19) = 25.5, *p*<.001, η*_p_^2^* = .57, and variant, *F*(1,19) = 5.1, *p*<.05, η*_p_^2^* = .21, were obtained. Participants showed a better performance for congruent (564 ms) than incongruent trials (593 ms), for the color (543 ms) than shape task (614 ms), and for East (575 ms) than West signs (582 ms). A significant two-way interaction of task x variant, *F*(1,19) = 13.2, *p*<.01, η*_p_^2^* = .41, showed that RT* was significantly faster for the East than the West sign variant in the shape task, *t*(19) = 4.5, *p*<.001, *d* = .94, but not the color task, *t*(19) = 1.1, *p*>.05, *d* = .27. No further two-way interactions reached significance (all *F*<2.1; all *p*<.05).

A significant three-way interaction of task x variant x congruency was observed, *F*(1,19) = 7.1, *p*<.05, η*_p_^2^* = .27. To resolve that interaction and to assess the influence of task and variant on the congruency effect, two further ANOVAs, splitting the three-way interaction by the factors variant and task, respectively, were carried out.

First, the split by variant showed for both variants main effects of task (East: *F*(1,19) = 14.9, *p*<.05, η*_p_^2^* = .44; West: *F*(1,19) = 37.1, *p*<.001, η*_p_^2^* = .66) and congruency (East: *F*(1,19) = 31.1, *p*<.001, η*_p_^2^* = .62; West: *F*(1,19) = 14.8, *p*<.05, η*_p_^2^* = .44). But only for the West signs, a significant interaction of task x congruency was observed, *F*(1,19) = 5.4, *p*<.05, η*_p_^2^* = .22 (see Figure S1), indicating a congruency effect in the shape task, *t*(19) = 4.1, *p* = .001, *d* = .91, but not color task, *t*(19) = 0.6, *p*>.05, *d* = .14. Hence, for West signs an incongruent color interfered with shape responses, but incongruent shape not with color responses. In contrast, there was no significant interaction of task x congruency for the East signs, *F*(1,19) = 0.1, *p*>.05, η*_p_^2^*<.01, because the congruency effect was significant in the shape task, *t*(19) = 4.7, *p*<.001, *d* = 1.05, and marginally significant in the color task, *t*(19) = 2.0, *p* = .063, *d* = .55. Thus, for East signs, color influenced shape responses, but the shape also affected the color responses to a certain degree.

These results are confirmed by splitting the three-way interaction by task indicating for the shape task a congruency main effect, *F*(1,19) = 26.1, *p*<.001, η*_p_^2^* = .58, and significantly faster RT* for East than West signs, *F*(1,19) = 20.2, *p*<.001, η*_p_^2^* = .52. In the color task, a significant interaction of variant x congruency, *F*(1,19) = 4.6, *p*<.05, η*_p_^2^* = .19, showed a tendency towards a congruency effect for East, *t*(19) = 2.0, *p* = .063, *d* = .55, but not West signs, *t*(19) = 0.6, *p* = .557, *d* = .14 (see Figure S2). Hence, shape responses were more efficient for the East signs, and in color responses, the shape of the East figures tended to interfere. There was no significant interaction of variant x congruency for the shape task, *F*(1,19) = 1.9, *p*>.05, η*_p_^2^* = .09, because the congruency effect was significant for East, *t*(19) = 4.7, *p*<.001, *d* = 1.05, and West signs, *t*(19) = 4.1, *p* = .001, *d* = .91. That means, color affected shape responses for both variants.

Some interactions were only marginally significant when *RT* instead of RT* was considered, particularly the two-way interaction of task x variant, *F*(1,19) = 4.2, *p* = .054, η*_p_^2^* = .18; the three-way interaction of task x variant x congruency, *F*(1,19) = 3.2, *p* = .089, η*_p_^2^* = .15; and the two-way interaction of variant x congruency for the color task, *F*(1,19) = 3.7, *p* = .068, η*_p_^2^* = .16.

In summary, the three-way interaction task x variant x congruency was driven by interference of incongruent color in the shape task, more efficient shape responses for the East sign, as well as more interference by East Ampelmännchen signs in the color task.

## Discussion

In the present study we examined cognitive conflict in a task with real-life signals for behavior. Specifically, we used East and West German versions of the stop/go Ampelmännchen pedestrian traffic signs. The experiment was intended to show whether (i) real-life objects such as traffic signs induce Stroop-like interference, (ii) color or shape of the signs produce more interference, and (iii) there are differences in interference produced by the West and East variants of the signs.

The results showed a congruency effect with a better performance for congruent than incongruent trials. Hence, the real-life objects induced Stroop-like interference. This result is not completely surprising, since the original meanings of the objects’ features, that is, color and shape, are obvious and well learned and thus, the incongruent information from the irrelevant feature interferes with the processing of the relevant feature. However, the observation that real-life stimuli produce effects similar to artifical stimuli is useful for the design of future experiments, as such natural stimuli tend to engage and motivate participants more strongly than artificial settings [12]. Using every-day stimuli may also be a step towards future experiments in virtual reality environments to test, for example, the efficacy of traffic signs in real-life settings.

Interestingly, in the present study, the congruency effect was influenced by the task as well as the variant of the sign. Regarding task differences, performance was better for the color compared to the shape task. Thus, the decoding of the meaning of the signs’ color appears to be easier. The reason may be that some stimulus attributes such as color are more effective in guiding visual attention than others, such as shape [13], [14] and the colors used may be highly overlearned in relation to traffic situations. In addition, significant interference was only observed in the shape task, that is, when sign shapes were paired with incongruent rather than congruent color. However, shape did not significantly interfere with the color task (apart from a trend for an effect for East signs as discussed below) suggesting that responses to sign color were more robust than responses to shape [15]. The result resembles the Stroop effect, where interference was seen for naming the ink color, but not reading of a color word, suggesting that reading is more automated and robust than color naming [10]. Here, color processing appeared more effective than shape processing, and thus color interfered with shape responses. Regarding real-life situations, our results on the one hand imply that color is the most salient information for pedestrian traffic lights and that the figure shape information may not be essential. On the other hand, color as a single feature would make the discrimination difficult for color-vision impaired people. One can argue that the combination of color and location (red at the top, green at the bottom) is sufficient for all pedestrians, as is the case for vehicular traffic lights. But the perception of the location is particularly affected by environmental conditions like darkness or fog, and shape is more unambiguous additional information. Furthermore, as outlined in the introduction, the Ampelmännchen signs were created to distinguish traffic lights for pedestrians from traffic lights for drivers, in order to reduce confusion and the risk of accidents. Thus, the shape information usefully complements and supports the essential color information of the signs in order to enhance pedestrian safety.

Intriguingly, we found significant differences between East and West Ampelmännchen signs. As hypothesized, shape responses were significantly faster for East signs and, color responses were disrupted more strongly by incongruent East than West figures. These observations cannot be simply explained by familiarity effects, because more participants reported to know the West than the East version. The results imply a comparatively more salient shape of the East German compared to the West German Ampelmännchen. Thus, the design of the East sign appears to be more expressive and the meaning of the figure more clearly apparent. In order to maximally gain the attention of pedestrians, the Ampelmännchen designer Karl Peglau created figures with visual features that are conspicuous and emotionally appealing to people, such as a hat, a bulbous nose and a plump, non-intimidating physique [2], [3]. The East sign also fills nearly twice as much colored space as the West sign. However, as our study showed, the different sizes of colored spaces alone do not explain the performance differences. Compared to the West figures, the more explicit shape of the East signs also leads to a larger amount of pixel differences between the East stop and go figures that may make them more effective. In addition, the shape of the East Ampelmännchen incorporates archetypical information of human behavior. The red figure with sidewards stretched arms clearly signals ‘stop’, while the green figure with its dynamic walking movements encourages to ‘go’. Such strong expressiveness is particularly missing from the West stop sign. Thus, it appears to be the shape design that explains the advantage of East versus West figures. More generally, one can consider a wide range of criteria for the effective visual design of traffic signs [16]. For example, such signs should attract attention, and their meaning as well as the expected response should be clearly understandable and intuitive, which are characteristics that appear more pronounced for the East than West Ampelmännchen. Desirable design criteria also include short reaction time, which in the present study was faster for East than West signs. However, some of the desired features may also be antagonistic. For instance, the requirement that a sign should be easily ignored when it is irrelevant for the expected response (“rejection” [16]), in order to minimize distraction, was better matched by the West than the East signs, since the East figures produced more interference and thus were harder to ignore. This interference appears to be a byproduct of the higher attractiveness of the East signs; and it may be generally impossible to design signs that fit all criteria perfectly. In any case, such criteria for effective traffic signs might provide a good starting point for future investigations.

One needs to point out that the current findings should not be over-interpreted, since the observed effects were small. Nevertheless, the effects we found may have implications for the design of effective traffic signals, and might also be used to compare further pedestrian signs, such as the ‘Euromännchen’, which was introduced as part of European-wide homogenization, or the ‘Ampelfrau’, an occasionally used female variant of the East Ampelmännchen [3], [17]. More generally, the used paradigm could also be expanded to other real-life situations, to assess, for example, the efficacy of signs depicting emergency exits or other critical pictograms. Finally, our findings of the high visual efficacy of the traditional East Ampelmännchen sign underline the practical utility of an East German icon.

## Supporting Information

Figure S1 **Impact of task on the congruency effect separately for East and West signs.**
(TIF)

Figure S2 **Impact of variant on the congruency effect separately for the color and shape task.**
(TIF)
